# Effect of Long-Term Biodegradable Film Mulch on Soil Physicochemical and Microbial Properties

**DOI:** 10.3390/toxics10030129

**Published:** 2022-03-07

**Authors:** Mingming Zhang, Yinghao Xue, Tuo Jin, Kai Zhang, Zhili Li, Chitao Sun, Qinghua Mi, Quanqi Li

**Affiliations:** 1 College of Water Conservancy and Civil Engineering, Shandong Agricultural University, Tai’an 271018, China; zmm@sdau.edu.cn (M.Z.); quanqili@sdau.edu.cn (Q.L.); 2 Rural Energy and Environment Agency, Ministry of Agriculture and Rural Affairs, Beijing 100125, China; xueyinghao@agri.gov.cn (Y.X.); stzzhbc@agri.gov.cn (T.J.); 3 Shandong Agricultural Environmental Protection and Rural Energy Station, Jinan 250100, China; zhangkai1987@shandong.cn (K.Z.); lizhili@shandong.cn (Z.L.); 4 Key Laboratory of Agricultural Film Application of Ministry of Agriculture and Rural Affairs, Tai’an 271018, China

**Keywords:** mulch materials, biodegradable film, soil physicochemical parameters, soil biological parameters, sustainability

## Abstract

Biodegradable mulches have become the focus of attention, as pollution caused by leftover plastic mulch material becomes increasingly severe. However, the impact of biodegradable mulches to the soil needs to be further investigated. An experiment was conducted to evaluate the impact of no-mulch, biodegradable film mulch (BM) and polyethylene film mulch (PM) on the soil’s physical, chemical and biological properties after six years (2013–2019) of mulching in garlic growing season in a garlic-maize rotation. Results showed that the soil bulk density of the 10–20 cm soil layer under BM decreased by 12.09–17.17% compared with that under PM. The soil total nitrogen content increased significantly by 14.75–28.37%, and the soil available phosphorus and potassium content increased by 64.20% and 108.82%, respectively. In addition, BM increased the soil’s microbial, soil urease, and soil catalase activities compared with those for PM. To sum up, BM can reduce soil bulk density, and long-term use of BM does not cause a decrease in soil nutrient content and microbial activity. On the contrary, it can improve soil quality. This study helps accumulate data for the environmental safety evaluation of BM and provides theoretical and technical support for the large-scale promotion of biodegradable mulches.

## 1. Introduction

In the middle of the twentieth century, the plastic industry emerged, and plastic film covering techniques followed. The mulching cultivation technique has been implemented in China since 1978 and has developed rapidly. Because of its pronounced effect of increasing and stabilizing production, it has been widely implemented and promoted [1]. Around the world, the global use of agricultural plastic was around 4.4 million tons in 2012 and reached 7.4 million tons in 2019 [2]. In 2020, China’s agricultural usage of mulches reached 1.357 million tons, covering an area of 1.74 × 10^7^ hm^2^ [3]. Owing to the promotion and popularization of mulching techniques, people have realized that plastic film mulch can preserve heat and moisture and prevent water evaporation, thus significantly increasing crop yield per unit area and making it possible to increase agricultural production and income [4,5,6]. However, the mass usage of polyethylene mulches has caused severe “white pollution” in agriculture and impeded the quality and safety of both cultivated land and rural environment. “White pollution” is a visual appellation of environmental pollution caused by waste plastics. Waste plastic thrown down to the fields, land and irrigation and drainage areas, scattered in rivers or hanging in shrub and tree branches became an important symbol of white pollution [7]. According to reports, the average amount of plastic film mulch residues on cultivated land in China is 34.0 kg hm^−2^, with a maximum of 317.4 kg hm^−2^ [8]. The incineration of film residues can produce organic pollutants such as furan and dioxins, which cause air pollution [9]. Plastic film fragments often mix with or remain in farmland to pollute farmland soil, and then pollute the marine environment through runoff [10]. Film residues floating in ditches, ponds and rivers will influence the quality of irrigation and drainage, cause water pollution and affect fish survival [9]. When plastic film mulch residue accumulates in the soil to a certain amount, it will reduce soil porosity and adversely affect soil structure and water movement, thereby inhibiting the distribution of crop roots, affecting the absorption of water and nutrients by the crops and limiting crop yields [11,12,13]. The research of Zumilaiti et al. [14] shows that an increase in the amount of plastic film residue decreases the growth rate of cotton root systems and the yield of cotton. When the residual film amount increased to 900 kg hm^−2^, the root length decreased by 33.7%, and the yield decreased by 22.2%.

In addition, plastic film mulch residue is one of the primary sources of microplastics that change the nature, function and biodiversity of the soil [15], thereby restricting the sustainable development of agriculture in China [9]. Promoting the application of fully biodegradable film replacement technology is a new route to solving farmland white pollution. At present, bioplastics account for about 1% of the global plastic production [16]. In 2018, the global annual production capacity of bioplastics was 2.112 million tons, of which biodegradable plastics accounted for 43.18% [17]. In 2020, the global capacity of biodegradable plastics reached 1.227 million tons, and agricultural film is one of the main target markets of biodegradable plastics [18]. Europe and Japan are the most advanced countries and regions for biodegradable plastic film development and application in the world. The market share has exceeded 10%, and the application proportion in some industries (such as vegetable planting in Japan) has reached more than 20% [19]. In China, agricultural plastic film is also one of the main downstream markets of degradable plastics. The Soil Pollution Prevention and Control Law of the People’s Republic of China [20] states that agricultural producers should be encouraged and supported to use biodegradable agricultural films.

To promote the wider application of biodegradable film, it is necessary to research the main crops that are mulched in specific areas. Garlic is an essential agricultural economic crop in China. In 2019, the sown area of garlic in China was 834,226 hm^2^, with a total output of 23.33 million tons, accounting for 75.89% of the total global garlic production (FAO). Good cultivation measures can provide a suitable environment for the growth and development of garlic, which is necessary to produce high-quality garlic sprouts and heads [21]. Studies have shown that mulching can significantly enhance the growth and development indicators of garlic, increase the quality of bulbs and bolting, and reduce the effects of freezing damage caused by abnormally low temperatures in winter [22]. To date, many biodegradable film mulch cultivation experiments have been carried out in garlic cultivation. The results show that biodegradable mulches were beneficial to the increase of yield [23].

Biodegradable films have been proven to be a promising alternative to polyethylene film in garlic mulching cultivation; however, there is a lack of research on the impact of biodegradable film on soil quality [21,22,23]. The effect of biodegradable film on soil quality will impact their future promotion and usage in agriculture. Soil quality not only affects farmland soil productivity but also determines the biological regulation and balance of the agroecosystems [24]. The deterioration of soil quality and health will have a significantly negative effect on many aspects of the ecosystem, so it is essential to monitor and assess the biodegradable film mulch effectiveness and long-term impact on soil health.

Soil indicators such as physical (e.g., bulk density, texture, and water holding capacity), chemical (e.g., pH, organic matter, total nitrogen, available potassium, and available phosphorus) and biological (e.g., enzymatic activities, microbial activity and microbial biomass) are helpful in the evaluation of soil quality [25,26]. Therefore, in this study, the soil’s physical, chemical and biological properties were investigated after the garlic was cultivated with different film mulch treatment. We hypothesized that soil quality would not be reduced by the use of biodegradable film mulch. Its effect on various soil parameters was tested and analyzed to provide a theoretical basis and technical support for the comprehensive promotion of biodegradable mulches, because they are significant in promoting a healthy soil environment and sustainable development in agriculture.

## 2. Materials and Methods

### 2.1. Experimental Site

The experiment was conducted at the Experimental Base of Shandong Agricultural University (117°32′ E, 36°13′ N), Lujiazhuang, Fangxia Town, Laiwu City, Shandong Province. This region has a warm temperate continental monsoon climate with four distinct seasons. The annual average air temperature is 11.0–13.0 °C, and the annual average rainfall is 760.9 mm. During the year, July and August are humid, September is semi-humid, and the other months are arid or semi-arid. Seasonal droughts occur frequently, with the highest probability in July, and heavy rains are mainly concentrated in summer, especially in July. Meteorological data regarding the study area were recorded between 2018 and 2019 (Table 1). Moreover, initial soil parameters were documented (Table 2). The data in Table 2 were recorded in September 2018, and the measurement method was the same as described in 2.3, below.

### 2.2. Experimental Design and Management

The experiment was conducted using a randomized block design with three treatments (CK, no-mulch; BM, biodegradable film mulch; and PM, polyethylene film mulch) and three replicates. The crop planting system at the experimental site of this study is garlic–maize rotation, with film mulching during the garlic growing season. No film mulching and no treatment were done during the growth period of summer maize. As of 2019, experiments had been conducted for six years (2013–2019). Biodegradable films [polybutylene co-adipate co-terephthalate (PBAT) + polylactic acid (PLA) content > 95%] were provided by the Agricultural Film Research Group of Shandong Agricultural University (2000 mm wide; colorless; the thickness is 0.008 mm in 2013–2016 and 0.006 mm in 2016–2019); the specification of the polyethylene films was 2000 mm × 0.006 mm, colorless. The coverage ratio of the mulches was 100%. The dimensions of the experimental plots were 25 m by 20 m. The total net study area was 432 m^2^. Each treatment has 3 plots with an area of 48 m^2^. The planting patterns were conventional flat planting. A local garlic variety, Jinxiang, was planted on 10 October each year with a planting density of 59.52 × 10^4^ plants⋅hm^−2^. Film mulching was carried out two days after sowing. The variety of summer maize is Zhengdan 958. It was planted on 4 June each year with a planting density of 7.94 × 10^4^ plants⋅hm^−2^. Garlic was harvested on 19 and 22 May in 2018 and 2019, and summer maize was harvested on 14 and 18 September in 2018 and 2019, respectively. The garlic was irrigated with 75 mm water before sowing, 105,000 kg⋅hm^−2^ of organic fertilizer and 750 kg⋅hm^−2^ of compound fertilizer (N-P_2_O_5_-K_2_O, 18-12-15; total nutrients ≥ 45%) were applied. Rotary tillage to 15–20 cm soil depth, Herbicide was sprayed after sowing. The garlic was irrigated for 75 mm at the bolting stage and bulb expansion stage, respectively, and a 750 kg⋅hm^−2^ compound fertilizer (N-P_2_O_5_-K_2_O, 18-12-15; total nutrients ≥ 45%) was added at the bolting stage. After garlic harvest, maize was directly sown without tillage, 750 kg⋅hm^−2^ of compound fertilizer (N-P_2_O_5_-K_2_O, 18-12-15; total nutrients ≥ 45%) were applied at the big bell stage, and 75 mm of irrigation was applied at the flowering stage. Except for different types of film mulch, other field management measures were the same and carried out following the local management regulations.

### 2.3. Experimental Methods

#### 2.3.1. Soil Samples

Soil samples were collected after the summer maize growing season (29 September 2018, and 30 September 2019) with three replicates. The five-spot-sampling method is adopted in each plot. That is, the midpoint of the diagonal is selected as the central sampling point, and then four plots on the diagonal with equal distance from the central sample spot are selected as sample spots. Soil samples from five spots were mixed as the final soil sample. The depths of the soil sample collection were 0–10 and 10–20 cm. Fresh samples were sieved to 2 mm, cleared of gravel and plant root residues. Soil samples were then divided into two parts, one was air-dried and the other was stored in ziplock bags at 4 °C.

#### 2.3.2. Soil Bulk Density

The core method [27] was used to determine the bulk density of soil at depths of 0–10 and 10–20 cm.

#### 2.3.3. Soil Chemical Properties

Soil samples were naturally air-dried and passed through a 2 mm sieve for the determination of the following soil nutrient parameters. Soil organic carbon (g⋅kg^−1^) was measured by the potassium dichromate oxidation method [28]. Soil total nitrogen (g⋅kg^−1^) was measured by the Kjeldahl procedure [29]. Soil alkali-hydrolyzable nitrogen (mg⋅kg^−1^) was measured using the NaOH-hydrolyzing, NH_3_-diffusing, H_3_BO_3_-absorption method [30]. As a plant nitrogen nutrient, alkali-hydrolyzable nitrogen content can accurately reflect the soil nitrogen supply level. Soil available phosphorus (mg⋅kg^−1^) was extracted with 0.5 mol⋅L^−1^ NaHCO_3_ and determined using the molybdenum-blue method [31]. Following the extraction with a 1 mol⋅L^−1^ NH_4_OAc solution, the soil’s available potassium was measured using a flame photometer [30].

#### 2.3.4. Soil’s Biological Properties

The soil’s microbial activity was measured using the CO_2_ release method. Five grams of fresh soil sample was cultured in a 280 mL reagent bottle at 22 °C for 24 h, and CO_2_ respiration was measured using GXH-3052L infrared CO_2_ gas analyzers (JUN-FANG-LI-HUA Technology-research Institute, Beijing, China). The soil urease activity (U⋅g^−1^), soil catalase activity (U⋅g^−1^) and soil phosphatase activity (U⋅g^−1^) were determined using soil urease kit (Solarbio, BC0120), soil catalase kit (Solarbio, BC0100) and soil phosphatase kit (Solarbio, BC0460) from (Beijing Solarbio Science & Technology Co., Ltd., Beijing, China) The soil urease, soil catalase and soil phosphatase activities were measured using the sodium phenol–sodium hypochlorite colorimetric method, ultraviolet absorption method and disodium phenyl phosphate colorimetric method, respectively. The determination methods are detailed in the manual.

### 2.4. Statistical Analysis

Data were statistically analyzed by a one-way ANOVA to determine differences between treatments after conforming to the basic assumptions of normality and homogeneity of variance. Statistical analyses were performed using the SPSS (Ver. 16.0, SPSS Inc., Chicago, IL, USA). The least significance differences (LSD) were used for differences among mean values at *p* < 0.05. Graphs were produced using Origin 2021.

## 3. Results

### 3.1. Effect of Biodegradable Film Mulch on Soil Bulk Density

**Figure 1:**
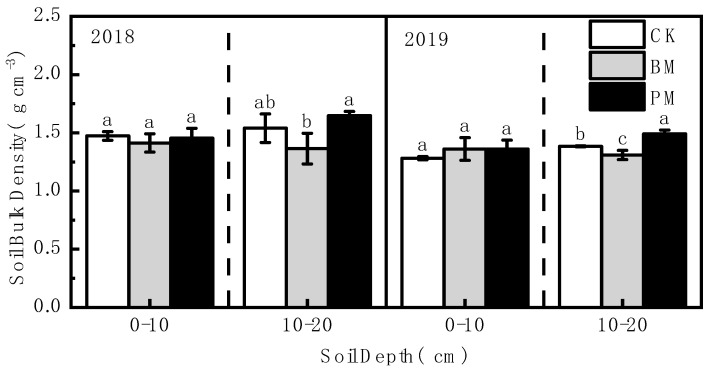
Effect of different mulch treatments (CK, no-mulch; BM, biodegradable film mulch; and PM, polyethylene film mulch) on soil bulk density. Values are means of three replicates ± standard deviation. Within each soil layer each year, the different letters above the columns indicate significant differences between treatments as tested by LSD (*p* < 0.05).

The soil bulk density of the 0–10 cm soil layer showed no significant difference among the different treatments in 2018 and 2019 (Figure 1). In the 10–20 cm soil layer, compared with polyethylene film mulch treatment, soil bulk density was significantly reduced under biodegradable film mulch treatment in 2018 and 2019, by 17.17% and 12.09%, respectively; compared with no-mulch treatment, soil bulk density was decreased by 11.38% and 5.33% in 2018 and 2019 under biodegradable film mulch treatment.

### 3.2. Effect of Biodegradable Film Mulch on Soil Nutrients

**Figure 2:**
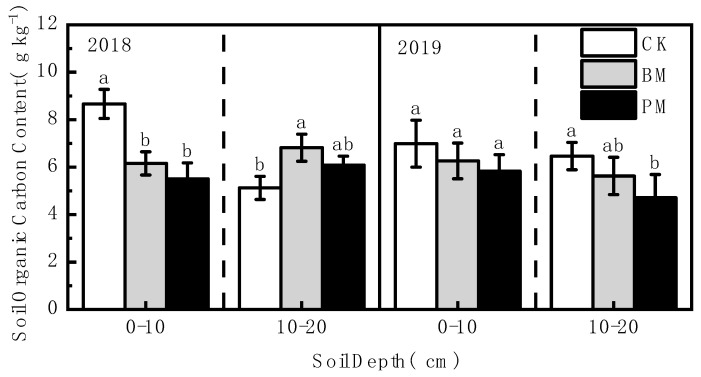
Effect of different mulch treatments (CK, no-mulch; BM, biodegradable film mulch; and PM, polyethylene film mulch) on soil organic carbon content. Values are means of three replicates ± standard deviation. Within each soil layer each year, different letters above columns indicate significant differences between treatments as tested by LSD (*p* < 0.05).

In 2018 and 2019, the organic carbon content of the 0–10 and 10–20 cm soil layers under biodegradable film mulch treatment was slightly higher than that under polyethylene film mulch treatment, but it did not reach a significant level. Except for the 10–20 cm soil layer in 2018, soil organic carbon content showed the following trend: no-mulch > biodegradable film mulch > polyethylene film mulch (Figure 2).

**Figure 3:**
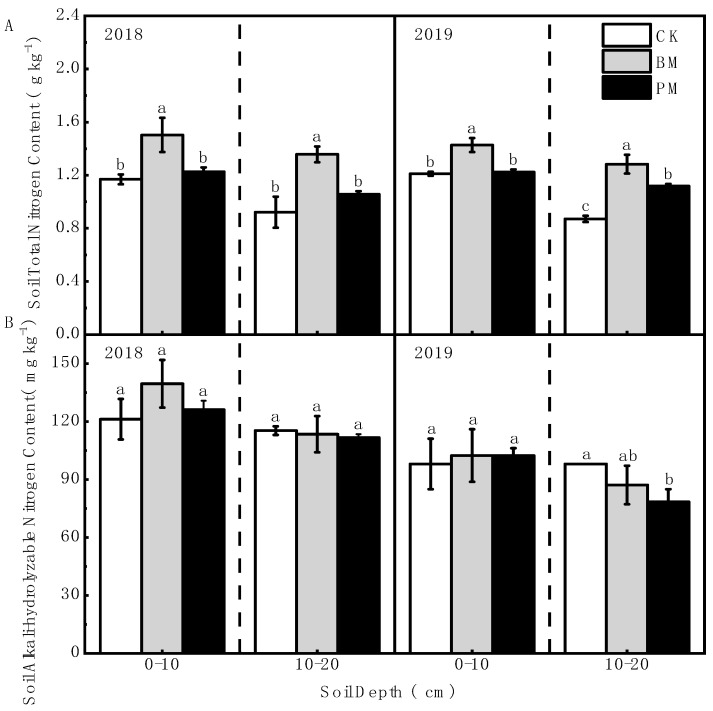
Effect of different mulch treatments (CK, no-mulch; BM, biodegradable film mulch; and PM, polyethylene film mulch) on the soil’s total nitrogen content (**A**) and the soil’s alkali-hydrolyzable nitrogen content (**B**). Within each soil layer each year, the different letters above the columns indicate significant differences between treatments as tested by LSD (*p* < 0.05).

The soil’s total nitrogen content under biodegradable film mulch treatment was significantly higher than those under polyethylene film mulch and no-mulch treatments. Compared with no-mulch treatment, the soil’s total nitrogen content under biodegradable film mulch treatment was increased by 28.53% and 47.34% in the 0–10 and 10–20 cm soil layers, respectively, in 2018, and by 17.85% and 47.35% in 2019; compared with polyethylene film mulch treatment, it was increased by 22.57% and 28.37% in 2018, and by 16.63% and 14.75% in 2019, respectively (Figure 3A).

There was no significant difference in the soil’s alkali-hydrolyzable nitrogen content between the biodegradable and polyethylene film mulch treatments. There was also no significant difference between the biodegradable film mulch and no-mulch treatments (Figure 3B).

**Figure 4:**
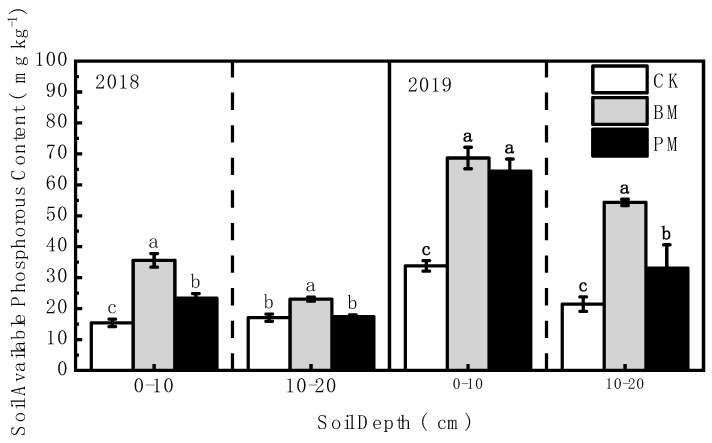
Effect of different mulch treatments (CK, no-mulch; BM, biodegradable film mulch; and PM, polyethylene film mulch) on the soil’s available phosphorous content. Values are means of three replicates ± standard deviation. Within each soil layer each year, the different letters above the columns indicate significant differences between treatments as tested by LSD (*p* < 0.05).

**Figure 5:**
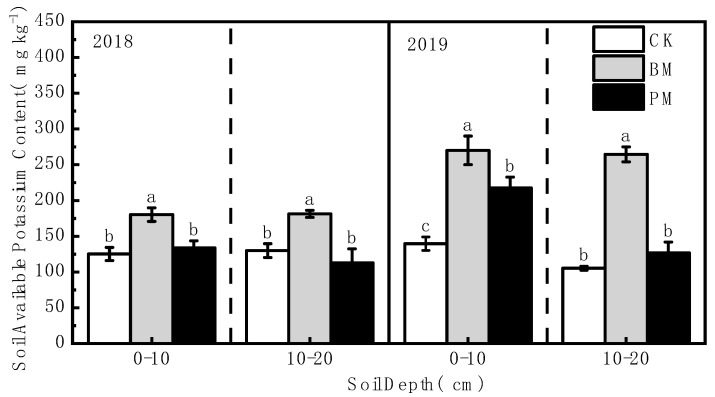
Effect of different mulch treatments (CK, no-mulch; BM, biodegradable film mulch; and PM, polyethylene film mulch) on the soil’s available potassium content. Values are means of three replicates ± standard deviation. Within each soil layer each year, the different letters above the columns indicate significant differences between treatments as tested by LSD (*p* < 0.05).

The soil’s available phosphorus and potassium content generally showed the following trend: biodegradable film mulch > polyethylene film mulch > no-mulch (Figure 4 and Figure 5). Compared with no-mulch treatment, the available phosphorus content in the 0–10 and 10–20 cm soil layers was significantly increased under biodegradable film mulch treatment, by 131.67% and 35.16%, respectively, in 2018, and by 103.16% and 153.38% in 2019. The soil’s available potassium content under biodegradable film mulch treatment was also increased significantly compared to that under no-mulch treatment, by 43.88% and 39.49%, and 93.32% and 151.11% in 2018 and 2019, respectively.

Compared with polyethylene film mulch treatment, the soil’s available phosphorus content was increased significantly under biodegradable film mulch treatment, by 52.14% and 32.57% in the 0–10 and 10–20 cm soil layers in 2018, respectively, and by 6.63% and 64.20%, in 2019. The soil’s available potassium content was also increased significantly under biodegradable film mulch treatment, by 34.91% and 60.47%, 24.04% and 108.82% in 2018 and 2019, respectively.

### 3.3. Effect of Biodegradable Film Mulch on Soil Microbial and Enzymatic Activities

**Figure 6:**
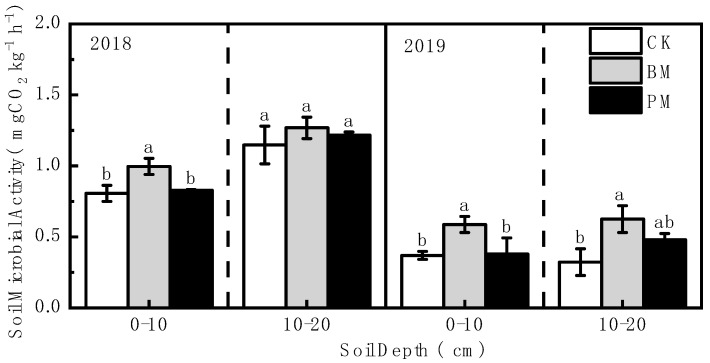
Effect of different mulch treatments (CK, no-mulch; BM, biodegradable film mulch; and PM, polyethylene film mulch) on the soil’s microbial activity. Values are means of three replicates ± standard deviation. Within each soil layer each year, the different letters above the columns indicate significant differences between treatments as tested by LSD (*p* < 0.05).

The soil’s microbial activity in each layer showed the following trend for both years: biodegradable film mulch > polyethylene film mulch > no-mulch (Figure 6). In the 0–10 cm soil layer, the microbial activity under the biodegradable film mulch treatment was significantly higher than that under the no-mulch and polyethylene film mulch treatments, increasing by 23.47% and 20.09%, respectively, in 2018, and by 58.97% and 55.00% in 2019. For the 10–20 cm soil layer, there was no significant difference in 2018; in 2019, the soil’s microbial activity under biodegradable film mulch treatment increased significantly by 94.12% compared to that under the no-mulch treatment.

**Figure 7:**
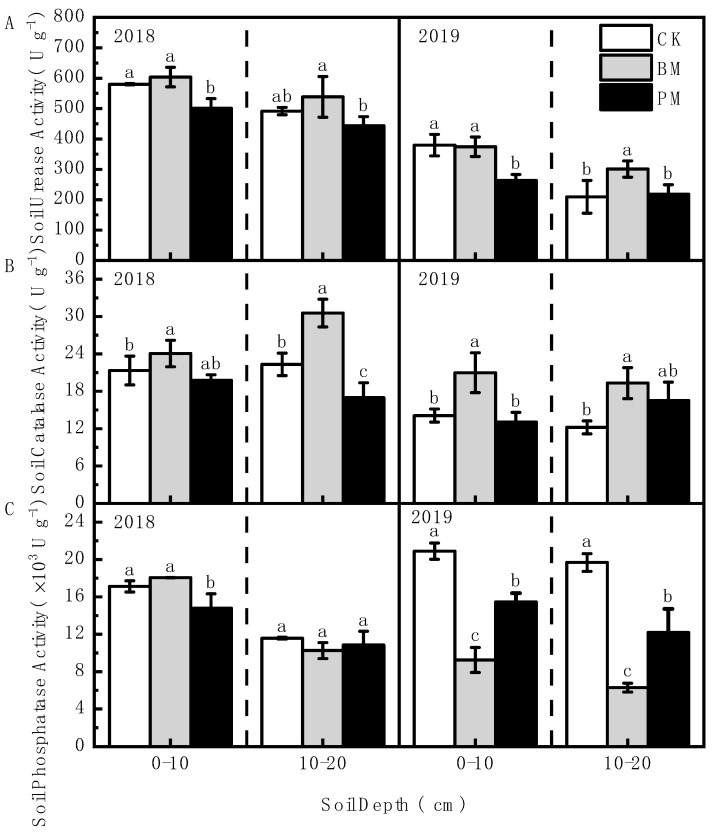
Effect of different mulch treatments (CK, no-mulch; BM, biodegradable film mulch; and PM, polyethylene film mulch) on the soil’s urease activity (**A**), the soil’s catalase activity (**B**) and the soil’s phosphatase activity (**C**). Values are means of three replicates ± standard deviation. Within each soil layer each year, the different letters above the columns indicate significant differences between treatments as tested by LSD (*p* < 0.05).

The results showed that the soil’s urease and catalase activity increased significantly under biodegradable film mulch treatment (Figure 7). Compared with no-mulch treatment, the soil’s urease activity increased by up to 43.80% under biodegradable film mulch treatment; compared with polyethylene film mulch treatment, the increase was up to 42.00%. Compared with no-mulch treatment, the soil’s catalase activity in the 0–10 and 10–20 cm soil layers was increased by 12.76–48.79% and 36.96–58.07%, respectively, under the biodegradable film mulch treatment; compared with that after polyethylene film mulch treatment, the increases were by 21.62–60.64% and 16.85–79.94%, respectively. Unlike the soil’s urease and catalase activity, the soil’s phosphatase activity under biodegradable film mulch treatment was generally lower than that under other treatments, especially in 2019.

## 4. Discussion

In recent years, biodegradable mulches have gradually become an effective measure to replace ordinary polyethylene mulches and solve the residual mulch pollution issue. Previous studies have shown that biodegradable film mulch can also improve the water and heat conditions of the soil’s plough horizon on farmland, and its moisture and heat preservation effects are similar to those of ordinary polyethylene mulches. It is feasible to replace common polyethylene mulches with biodegradable mulches for the cultivation of potatoes, cotton, peanuts and beets [32,33,34,35,36]. The effect of biodegradable mulches on soil health will impact their promotion and application in agriculture. Currently, there are only a few reports on the impact of degradable mulches on soil quality. Li et al. [37] studied the changes in soil quality after applying biodegradable mulches with different materials for 18 months, and the results showed that the impact was minor. Applying mulches for a short period may not be enough to observe the changes in soil quality. There have been relatively few studies on the impact of biodegradable mulches on soil quality after long-term application on farmland. The effects of six years of application of biodegradable mulches on the soil’s physicochemical and biological properties were investigated in this study. Compared with the no-mulch treatment, there is no apparent adverse effect; on the contrary, biodegradable mulches are beneficial to soil fertility.

Soil bulk density is a crucial index reflecting the soil’s physical quality. It affects soil–plant interaction processes, such as gas exchange, water availability, water infiltration, biological activity and plant rooting depth, thus affecting the soil’s function. The results from this study showed that biodegradable film mulch has no significant impact on soil bulk density compared with the no-mulch, except for the 10–20 cm soil layer in 2018. The results are almost consistent with those of Gao et al. [8], who reported that no significant difference was observed with no residual film and residual biodegradable film treatments. This is because biodegradable plastic film can degrade rapidly, and the residue of biodegradable plastic film in the soil is reduced to a shallow level. The components released during the biodegradation process accumulate and continue to biodegrade until they are fully mineralized into CO_2_ and H_2_O [38]. Moreover, the soil’s physical indicators are usually considered “slow-change” indicators [26]. Therefore, biodegradable plastic film treatment has a minor effect on soil bulk density. In the present study, the soil bulk density with the biodegradable plastic film was significantly lower than that of polyethylene plastic film treatment in a 10–20 cm soil layer. It has been reported that the accumulation of residual polyethylene film in soil can reduce the soil’s porosity and pore connectivity [7], thus interfering with the soil’s structure [12]. Gao et al. [8] also reported that an increased bulk density was observed in residual polyethylene film treatment compared to no residual film and residual biodegradable film treatments.

Apart from soil bulk density, soil nutrients were also influenced by biodegradable mulches. In this study, the contents of total nitrogen, available phosphorus and available potassium in the soil were all increased under biodegradable film mulch treatment. Generally, plastic films serve to conserve the soil’s moisture and regulate soil temperature [39,40]. Increasing either soil temperature or moisture can enhance soil nutrient mineralization [41], so soil nutrients were also influenced by mulching. Studies have shown that biodegradable mulches are rich in organic carbon [28]. Upon addition to the soil, they have a positive effect on the soil’s carbon storage and can increase its organic carbon content [42]. Zumstein et al. [43] found that soil microorganisms use the carbon of PBAT to obtain energy to increase the soil’s carbon inventory. However, in this study, there was no significant difference in the organic carbon content between biodegradable film mulch and polyethylene film mulch treatments, and the carbon content under mulch treatment was generally lower than that under no-mulch treatment. This is because the effect of mulch treatment on organic carbon is a balance among root growth, secretion increase, microbial decomposition and CO_2_ loss [44,45,46]. In addition, the increase in temperature and humidity caused by mulching may stimulate the soil’s organic carbon mineralization, and studies have shown that mulching will increase the decomposition of the soil’s organic carbon at the later stage of crop growth [47,48]. Therefore, there have been positive and negative results from studying the changes in the soil’s organic carbon content under mulching. Some reports state that mulching can increase the soil’s organic carbon contents [49], whereas some claim that there is no change [50] or there is a decrease [51].

Soil mulching treatments also affected soil microorganisms and the soil’s enzymatic activities. A few research reports show that microbial abundance, respiration and enzymatic activity were increased under biodegradable film mulch treatment, as compared with that under polyethylene film mulch treatment [37,52,53,54], which indicates that biodegradable mulches do have a certain impact on microbial activity and the soil’s enzymatic activity. This is consistent with the results of this study, in which the soil’s microbial activity, urease activity and catalase activity increased significantly under biodegradable film mulch treatment compared with that under polyethylene film mulch treatment. Averaged over the two years, in the 0–10 cm layer, the soil’s microbial activity, urease activity and catalase activity were increased by 37.55%, 10.24% and 10.81%, respectively; in the 10–20 cm layer, they were increased by 17.23%, 10.64% and 39.97%, respectively. Studies have shown that exogenous organic substances in agricultural soil would affect the complexity and metabolic processes of the soil’s microbial networks [55]. For the biodegradable plastic film, the released monomers during degradation are to be used by microorganisms to grow, increasing microbial biomass [38]. Biodegradable film mulch can also improve the soil’s microclimate. Favorable water and temperature conditions under the mulches affect the plant root system, generally stimulating root development and increasing root secretion [50,56,57], and these changes all regulate microbial and enzymatic activity.

Along with the increase of the variety, commercialization and availability of biodegradable plastic film, as well as the increased awareness of environmentally sustainable development, there has been a rising interest towards a deeper understanding of the application effect and the influence on the agroecosystem of biodegradable plastic film. In terms of whether biodegradable plastic film can be a competitive alternative to polyethylene plastic film, Sintim and Flury [58] proposed several standards, including six requirements on microclimate, mechanical operability, integrity of planting season, degradability, environmental impact and economy. Ecotoxicity assessment of the biodegradable plastic film and its components has attracted much attention, but this research is still in its infancy. The understanding of the effect of biodegradable plastic film on soil environment is mainly based on short-term experiments for several months [37,59]. The long-term impact of the repeated use of biodegradable plastic film on agricultural soil health in the whole crop season and year still needs to be studied. In addition, biodegradation is governed by many biotic and abiotic factors under field conditions. It may not be possible to control biodegradation in natural environments, so how to accelerate the degradation of biodegradable mulch in the soil also needs to be studied. At present, some studies have made corresponding progress. For instance, Fontanazza et al. [60] isolated and identified the mesophilic bacterium *Pseudomonas putida* from soil particles and found that the bacterium *Pseudomonas putida* was responsible for the degradation of biodegradable plastic. This may lead to the use of plastic-degrading microorganisms for amendments to the soil where plastics need to be degraded or to accelerate the degradation process. At the same time, the migration of compounds and chemicals released from the biodegradable plastic film from agricultural systems to other systems, such as transportation and accumulation in food webs, should also be considered. All these researches will benefit the sustainability of the agroecosystem to a great extent.

## 5. Conclusions

The analysis of a soil’s physical and chemical properties, and the microbial and enzymatic activity under different mulch treatments for 6 years, showed that the soil bulk density under biodegradable film mulch treatment was lower than that under polyethylene film mulch and no-mulch treatments. The long-term usage of degradable mulches did not cause soil quality degradation; on the contrary, it increased the content of the soil’s total nitrogen, available phosphorus and available potassium, and improved the soil’s microbial activity, urease activity and catalase activity, which are conducive to soil fertility improvement. Therefore, we believe that from the perspective of protecting soil environments, biodegradable mulches are promising and sustainable alternatives to polyethylene mulches.

## Figures and Tables

**Table 1:** Monthly meteorological data in 2018 and 2019.

Month	Rainfall (mm)		Temperature (°C)	
	2018	2019	2018	2019
January	6	6.4	−2.7	−4
February	3	10.5	0.5	6
March	26	16.6	8.9	15
April	62	34.3	15.7	20
May	62	50.2	21	28
June	137	99	25.9	32
July	233	222.6	27.6	32
August	245	150.7	27	28
September	66	70.2	21.1	27
October	11	30.8	14.5	20
November	31	21.4	7.8	13
December	15	8.9	−0.6	6

**Table 2:** The initial soil parameters of the 0–20 cm soil layer.

Soil Texture	Percentage of Particle Content (%)			pH	Soil Bulk Density (g⋅cm^−3^)	Organic Carbon Content (g⋅kg^−1^)	Total Nitrogen Content (g⋅kg^−1^)	Available Nitrogen Content (mg⋅kg^−1^)	Available Phosphorus Content (mg⋅kg^−1^)	Available Potassium Content (mg⋅kg^−1^)
	Clay 0–2 μm	Silt 2–50 μm	Sand 50–2000 μm							
Sandy loam	2.49	24.09	73.42	7.9	1.51	6.89	1.05	118.26	16.22	127.67

## Data Availability

The data presented in this study are available on request from the corresponding author.
